# Grand Challenges in Public Health Policy

**DOI:** 10.3389/fpubh.2015.00029

**Published:** 2015-02-18

**Authors:** Tarun Stephen Weeramanthri, Ross Stewart Bailie

**Affiliations:** ^1^Department of Health, Public Health and Clinical Services Division, Government of Western Australia, Perth, WA, Australia; ^2^Centre for Primary Healthcare Systems, Menzies School of Health Research, Charles Darwin University, Brisbane, QLD, Australia

**Keywords:** public health, policy, challenges, ethics, definitions, epidemiology, partnerships, international

## Introduction

Fifteen years into the twenty-first century, the challenges for public health may have changed but have not diminished. Complacency, systems inertia, and national self-interest remain powerful barriers to global and universal health improvement.

As joint chief editors for this new specialty journal, we lay out an ambitious mission: to work with the editorial board and colleagues worldwide to create the pre-eminent open access journal for public health practitioners working in policy and practice.

The aim of this article is twofold: to scope out the breadth of topics that may be covered in the journal, and to reflect on the kind of difficult but essential conversations that may be possible in such an enterprise. We hope to encourage diverse contributions to the journal that reflect deeply on the contingent nature of public health policy as it is put into practice in a range of contexts and settings across the world, as well as the commonalities that underpin it.

This article is structured into four parts: a brief outline of historic and current practice; a discussion of definitional and ethical issues in public health; a reflection on strengths and weaknesses as we see them; and, finally, a set of grand challenges that we hope to address in this journal, which are central to the grandest public health mission of all – changing the world for the better.

## Public Health – Then and Now

History has shown us the dramatic improvements in health outcomes and life expectancy that follow improvements in housing, sanitation, drinking water, education, employment, working conditions, food supply, transport infrastructure, and other social determinants of health. Over time, these measures become incorporated and normalized in legislation of various forms (health, occupational health, welfare, environmental, etc.), and later taken for granted and almost invisible in societies (1). The tragedy is that such basic supports for health are still not present in all countries. In addition, the current Ebola outbreak in West Africa has served to remind us of the importance of universal access to basic primary health care, and how the lack of investment in such care can threaten the economic livelihoods of nation-states (2).

We are also in the midst of a number of concurrent societal revolutions that impinge directly on health care and public health. As life expectancy increases, the population ages, technology improves, and the burden of chronic diseases grows, so spending on health care as a proportion of GDP tends to rise much faster than non-health areas of expenditure, and in a potentially unsustainable way (3).

Partly because of its historical origins, public health remains part of the health care system, though its success relies heavily on partnerships with other sectors. It is often seen as the “poor cousin” of health and medical care, with a relatively small level of investment compared to its economic returns (4), which are often longer-term and indirect, and therefore “invisible” to decision-makers.

Fast, global travel has been with us for some decades, while urbanization now sees the majority of the world population living in cities for the first time in human history. The speed of change is particularly evident in information technology. Mobile phones with spatial positioning capability and internet access are now seen even in the midst of extreme poverty, with current and potential applications for personalized health maintenance and organized health care (5).

New commercial entities and not-for-profit organizations are fast entering the personal health market and have started competing to provide what have been traditional government functions in many countries (both health care services and other areas such as health promotion).

Traditional public health practice has had a central reliance on data and information, and the core discipline of epidemiology, in order to inform health policy and priority-setting, drive health improvement across whole populations, and target disadvantaged populations (6). The fight for data – to complement experience and counter myth and disinformation – is at the heart of public health. Some public health principles are counterintuitive rather than common sense; try explaining to a lay audience the benefits of a “population strategy” for prevention over a “high-risk” approach (7).

Other core activities of public health include community education, outbreak investigation and communicable disease control, risk factor and disease surveillance, screening, development and implementation of public health interventions, evaluation, and research. Since 1970s, New Public Health has also emphasized community engagement, health promotion, inter-sectoral partnerships, and advocacy, and encouraged “settings” and “whole of life” strategic approaches (8). The importance of the partnerships approach is highlighted by the international experience with HIV/AIDS since 1980s (9).

## Definitional and Ethical Issues

Definitions of public health abound, from the “organized response by society to protect and promote health, and to prevent injury, illness, and disability” (10), to aiming to “provide the necessary conditions for a population to be healthy” (11). Whatever the merits of various classification systems, no single definition has been particularly effective in galvanizing community and political support for investment in public health, or even in convincing health colleagues of its place or importance.

We prefer as a starting point a clear description using six key words (see italics) of the three core functions of public health; namely, to *promote health* in the community, *prevent disease* before it occurs, and *manage risk*, either natural or man-made. There is a constant interplay between individuals and their physical, social, and cultural environments. When seen in this way, the diversity of public health activities, the A–Z of public health (Asbestos to Zoonoses), can be categorized and managed.

At its heart, public health is a conversation society has, and will continue to have, about the balance between individual rights and the “common good” or “public interest” (12). As such, it is as much a branch of public policy as a branch of medicine or health science. This conversation evokes questions like “How might we discern the common good, and who can speak for it? Is there a consensus about the rights and benefits to which citizens should be entitled, and the obligations of citizens to society, the state and one another?” Public health professionals and policy-makers have principles, which we call on, such as the “precautionary principle,” “proportionality,” or “intergenerational equity,” but these are not complete philosophical answers, nor can they ever be.

## Strengths and Weaknesses

It is important for the public health community to counter the “invisibility phenomenon” mentioned earlier by continuing to emphasize the dramatic cost-effectiveness and lives saved from traditional public health measures. These include sanitation, clean water, hygiene promotion, immunization, tobacco control, fluoridation, maternal and child health services, road safety programs, organized cancer screening, access to essential drugs, sexual health and reproductive services, and needle and syringe exchange programs (13).

But, we also have to be realistic about weaknesses in the current approaches, and new barriers to health improvement. Apart from lack of investment, workforce needs are difficult to define given the diversity of roles and functions (14). Workforce planning in public health is rudimentary and the lack of planning could be detrimental in a generation’s time as global burden of disease shifts further from communicable to non-communicable diseases. Part of that shift is driven by commercial vectors – the Big 3 commercial interests – tobacco, alcohol, and fast food companies – with their growing network of connections from the corporate board table through to media, marketing, and retail interests. They are powerful, well funded opponents, who will often misrepresent any public health proposal from government as indicative of the “nanny state” (15).

## Grand Challenges

Public health policy and practice is constructed and realized at many levels simultaneously – local, regional, national, and international. All are important, require different skills and should be linked to maximize effectiveness. As the context for public health changes, institutions will need to be reinvented and new networks formed to bridge the gap between these levels, and to stimulate new communities of learning and action. We hope that this new specialty journal will constitute such a new reflective community. In particular, we want to see contributions from people in the field, who do not see themselves as “academic,” as well as from colleagues based in learning institutions.

This new community of practice should provide a welcome challenge for many traditional experts in public health. New forms of digital communication mean the public are joining the societal discussion, indeed often instigating it, at an earlier stage, along with a broad range of other stakeholders. New forms of grass-roots activism and advocacy are sprouting next to traditional public health and health promotion programs, and are attracting considerable media and public attention. This may be a new opportunity for health promotion partnerships, but such movements may also distort traditional priorities for investment of scarce public resources, especially if they are co-opted by commercial interests (16).

The success of public health policy can only be measured by what happens in practice, with the other contextual “P” of public health being politics. And since anything inherently political is always controversial, public health practitioners have to be prepared to defend and argue their points of view, including in this journal and with each other, and critically analyze and communicate the success of their programs to a variety of health and non-health stakeholders.

There are strong links between public health and other agendas that focus on human dignity, gender equity, and human rights (17). Sensitivity to cultural and linguistic factors is crucial in public health policy and practice, starting at the local level, while an analysis of the distribution of power and resources across society is essential to understanding outcomes at a national and international level. Some groups will always struggle to voice their concerns (e.g., homeless or prison populations) and will need help from outside advocates. Essential health care and public health services are not a luxury, nor can their delivery as a public good to vulnerable populations be left to the market or outsourced entirely to the private sector.

We believe that the true potential of public health will only be realized with a full “head, hearts, and hands” approach. Public health is a knowledge industry, driven by the importance of ideas (“the head”), and delivered by the hands of its diverse workforce. But, at its heart, it is underpinned by a passion for people and a commitment to change. Contributors to this new specialty journal should be unafraid to demonstrate that passion and commitment in their writing.

This new community of public health policy and practice will also seek to draw on different ways of thinking about our world, through the promotion of interdisciplinary approaches to public health. For example, social and behavioral scientists – often not at the table when public health interventions are being planned, delivered, and evaluated – can offer crucial insights on the importance of cultural and group context, and the mediating effects of individual attitudes and beliefs on subsequent habits and choices. Similarly, economists, planners, environmental scientists, and the like offer crucial perspectives and should be integrated into public health policy and practice networks. More fundamentally, other disciplines are required to design whole system responses to complex issues (18).

All the above considerations determine what we see as the grand challenges in public health policy, and how we might envisage meeting them. We have grouped them under 10 themes:

1. The fundamental determinant of sustainability – a healthy planet and environment. Public health must be at the forefront of action to deal with traditional environmental risks, and to mitigate and adapt to climate change; and good scientific policy must drive both analysis and actions.
2. Response to population changes. As life expectancy increases, and the burden of chronic, non-communicable diseases rises globally, there is a twofold challenge for policy and action. How best to promote wellbeing and healthy living/aging, and to combat commercial forces driving unhealthy behavior (particularly tobacco, diet, physical inactivity, obesity, alcohol misuse, and gambling).
3. Creating a true global health system that strengthens basic national health care systems, bolsters existing international institutions, and creates new networks and communities of practice between developed and developing countries – so that, in particular, nation-states can respond in a timely and coordinated way to new regional and global threats.
4. Continue to make the case for the value of investment in public health, and develop more explicit accreditation, performance, and workforce planning frameworks that show how resource use is and will be linked to quality of service, and specific population-level outcomes.
5. Work with other sectors, media, and advocacy coalitions on what we term “eternal challenges”: poverty, equity, powerlessness, discrimination, and stigma.
6. Strengthen linkages between public health and clinical care systems to help make national health systems more affordable and sustainable.
7. Realize the opportunities new technologies provide to more precisely target populations and improve effectiveness of interventions.
8. Actively look for ways to harness greater public involvement in public health decision-making, including through the use of social media, online consultation, and greater public access to government data.
9. Develop a more comprehensive ethical and regulatory framework for public health that moves beyond individualism, promotes equity and public good considerations, teases out terms like the “precautionary principle” and “intergenerational equity,” while at the same time respecting individual human rights and the opportunity for individual advancement.
10. Embed research and innovation, and interdisciplinary partnerships between health, social and behavioral scientists, economists, and others, as a fundamental component of any knowledge system, not as an afterthought.

As readers, writers, and editors in this new community of practice, there will be a diversity of views expressed on any difficult or controversial topic. The editorial board will look at innovative ways to encourage such debates to be held in a robust but respectful way. Editors will also be aware of our own position in the debates; not necessarily high-minded, virtuous experts and disinterested arbiters of the common good, but sometimes partisan, conflicted and in the fray. At all times, however, we will apply the principles of good scientific peer review, be transparent in our dealings and be accountable for our views, reviews, and decisions.

This set of grand challenges, and the way we meet them as practitioners, policy-makers, journal readers, and citizens, will help define a new generation of public health leaders. Trained in core traditional disciplines, and also more eclectic in practice and theory than current leaders, comfortable with uncertainty, committed to teamwork, partnerships and public dialog, and willing to try and fail and try again.

## Conflict of Interest Statement

The authors declare that the research was conducted in the absence of any commercial or financial relationships that could be construed as a potential conflict of interest.
